# Editorial: Genetic improvement of *Triticeae* crops based on high-throughput phenotyping: Molecular design for yield, resistance and tolerance

**DOI:** 10.3389/fpls.2023.1165461

**Published:** 2023-03-09

**Authors:** Xiaoli Fan, Pengtao Ma, Fa Cui, Yunfeng Xu, Shuyu Liu

**Affiliations:** ^1^ Chengdu Institute of Biology, Chinese Academy of Sciences, Chengdu, China; ^2^ School of Life Sciences, Yantai University, Yantai, Shandong, China; ^3^ School of Agriculture, Ludong University/Key Laboratory of Molecular Module-Based Breeding of High Yield and Abiotic Resistant Plants in Universities of Shandong, Yantai, China; ^4^ Agricultural Genomics Institute at Shenzhen (AGIS), Chinese Academy of Agricultural Sciences (CAAS), Shenzhen, China; ^5^ Texas A&M AgriLife Research, Texas A&M University System, Amarillo, TX, United States

**Keywords:** molecular design, high-throughput phenotyping, stress tolerance, yield, *Triticeae* crops

## Introduction

*Triticeae* are the foremost staple foods worldwide and also the most widely cultivated crops. Their resistance to biotic and abiotic stresses is always important during genetic improvement and thus greatly influences their adaptability and cultivation ranges. However, still-evolving pathogens and pests, harsh weather, reduced resources, etc., all threaten sustainable crop production, making resistance and tolerance improvement the most important concerns in current *Triticeae* breeding programs. Meanwhile, with the global population increasing, crop production is facing great growth challenges to meet food security demands, further emphasizing the importance of overcoming these adverse conditions that jeopardize crop production. Fortunately, in recent years, with the accelerated progress of omics research, resistance/tolerance-related genes have been rapidly discovered, and associated diagnostic single nucleotide polymorphisms have been effectively developed, making the application of germplasm resources for molecular design breeding more efficiently, and providing sustainable sources of resistance and yield improvement for *Triticeae* crops to ensure future food security.

In relation to this topic, a total of 140 authors contributed their recent advances in disease resistance, stress tolerance and yield performance studies on *Triticeae* crops, which are presented in 13 research articles.

## Biotic stress response in *Triticeae* crops

Powdery mildew, a globally epidemic disease in *Triticeae* crops caused by the biotrophic fungus *Blumeria graminis* f. sp. *tritici* (*Bgt*), can severely affect yield and quality. Even though more than 100 powdery mildew (*Pm*) genes/alleles have been found in *Triticeae* crops, only a few of them have been applied in wheat improvement (Han et al., 2022). These crops are facing strong selection pressure, and continual identification and utilization of *Pm* genes/alleles from various germplasm resources are needed for rational deployment. Li et al. reported a novel *Pm* gene, *PmSN0293*, on wheat chromosome 6A, putatively from *Thinopyrum ponticum*, and developed two wheat–*Th. ponticum* introgression lines carrying this new *PmSN0293* and the previously reported *Pm2* and *Pm52*, which exhibited excellent application potential in wheat breeding programs. Jin et al. identified a new splicing variant of Pm4, PmYAV, in synthetic hexaploid wheat, which was developed by hybridization of diploid Aegilops and tetraploid wheat, and four molecular markers available for marker-assisted selection (MAS) were screened. Liu et al. identified a major quantitative trait locus (QTL) *QPm.cas-7D* for adult plant resistance to wheat powdery mildew in a well-known wheat–*Agropyron cristatum* introgression line, PuBing3228. It was subsequently deduced that QPm.cas-7D was Pm38 and that a 3-bp InDel between resistant and susceptible haplotypes was responsible for the resistance. Qiu et al. identified a resistant haplotype carrying the Pm locus, PmH962, which was confirmed to be the reported Pm5e and had no negative effect on wheat agronomic performance.

In addition to powdery mildew, resistance to other destructive diseases, such as leaf rust and stem rust, were also presented. Zhang et al. applied 112 wheat accessions introduced from the U.S. National Plant Germplasm System to evaluate resistance to the Chinese predominant races causing leaf rust and their resistance genes in order to explore more effective resistance resources for overcoming wheat leaf rust. Kataria and Kaundal performed a comparative analysis of protein-protein interactions (PPIs) between two pathogen races, *Pgt* 21-0 and *Pgt* Ug99, both causing wheat stem rust, and elucidated the functional differences between these two races, thus providing the strain-specific information for the development of durable, disease-resistant crop lines.

In addition to the genetic basis for pathogen stress, Luo et al. also reported the fine mapping study of a *Hairy glume* gene, responsible for trichomes on wheat glumes, which is largely involved in resistance to various biotic and abiotic stresses, as well as in defense to against insect pests.

## Abiotic stress response in *Triticeae* crops

Natural cultivation conditions, such as light, nutrients, water, and salinity, determine crop adaptability and affect their yield and quality. Yang et al. discovered that low-light stressed wheat could alter its pollination type to enable outcrossing with heterologous pollen by increasing lemma and glume angles, which finally compensated for the 2.1–18.0% loss in grain number. Liu et al. analyzed the transcriptional mechanism of the response to low-nitrogen stress of a previously located major stable QTL for wheat root growth, QMrl-7B, and found genes encoding NO^3-^ transporters, etc, composing the complex regulatory network for root determination. Li et al. also performed the transcriptome profiling of the well-known transcription factor gene family, WRKYs, in Tritiprum and the response of TtWRKY256 to salt stress. Moreover, considering that the chlorophyll content could directly impact photosynthesis and affect crop health, Wang et al. proposed an unmanned aerial vehicle (UAV)-based approach to rapidly and efficiently predict chlorophyll content under irrigation and drought stress to provide insights into the capacity of UAV-based remote sensing for phenotyping to improve crop breeding.

## Yield-related trait response to stresses in *Triticeae* crops

Maintaining yield performance under various unfavorable conditions is the main concern for breeders during genetic improvement of resistance or tolerance to both biotic and abiotic stresses (Deng et al., 2017). Wang et al. studied a set of 18 yield-related and agronomic traits in wheat under different irrigation regions by QTL analysis to provide insight into the genomic regions contributing to high yield in water-limited conditions and reported a novel QTL stably controlling wheat kernel length under all tested environments, facilitating the future MAS for pyramiding the favorable loci for high-yield improvement. Zhang et al. identified three QTLs for wheat spikelet nodes per spike and investigated incorporation of their favorable alleles across different wheat agroecological production zones of China, such as Chengdu of the Southwestern Wheat Zone and Yuncheng and Linfen of the Huai River Valleys Facultative Wheat Zone, where the natural environmental factors were significantly different, advancing our understanding of the genetic basis of natural variation in spikelet development and its adaptation to environmental change.

In summary, innovative and attractive advances have been made in understanding both biotic and abiotic stress responses of *Triticeae* crops and their contribution to yield potential, which will facilitate wheat genetic improvement and food safety.

## Author contributions

All authors listed have made a substantial, direct, and intellectual contribution to the work and approved it for publication.
